# Supplementary figures and images for: Munc13 supports fusogenicity of non-docked vesicles at synapses with disrupted active zones
Source: eLife. 2022 Nov 18;11:e79077. doi: 10.7554/eLife.79077 (PMC9822248; doi:10.7554/eLife.79077)

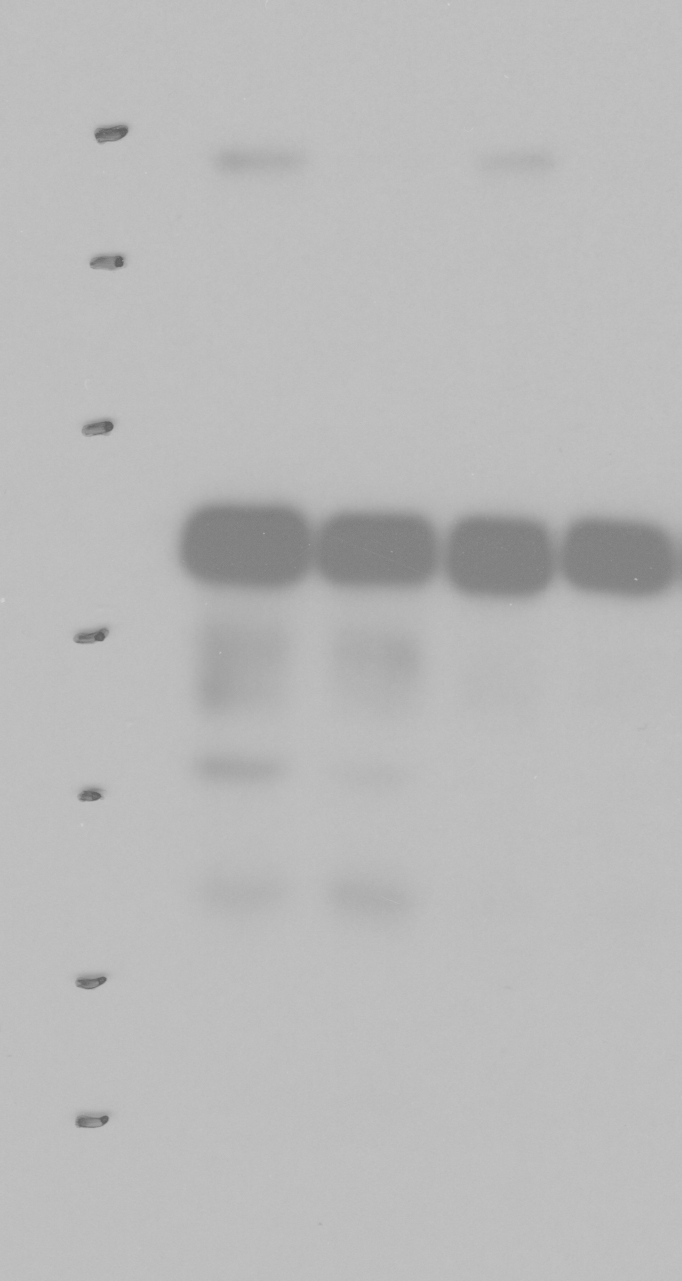

Supplement: Figure 1—figure supplement 3—source data 1. — (A) Original scans (1 s and 30 s) of Western blots shown in Figure 1—figure supplement 3C. (B) Raw grayscale scans (left) and brightness- and contrast-adjusted scans (right) of Western blots shown in Figure 1—figure supplement 3C. (C) Original scans (5 s and 1 min) of Western blots shown in Figure 1—figure supplement 3F. (D) Raw grayscale scans (left) and brightness- and contrast-adjusted scans (right) of Western blots shown in Figure 1—figure supplement 3F. [file elife-79077-fig1-figsupp3-data1.zip › Figure 1-figure supplement 3-source data 1/Figure 1-figure supplement 3-source data 1A-1s.tif]

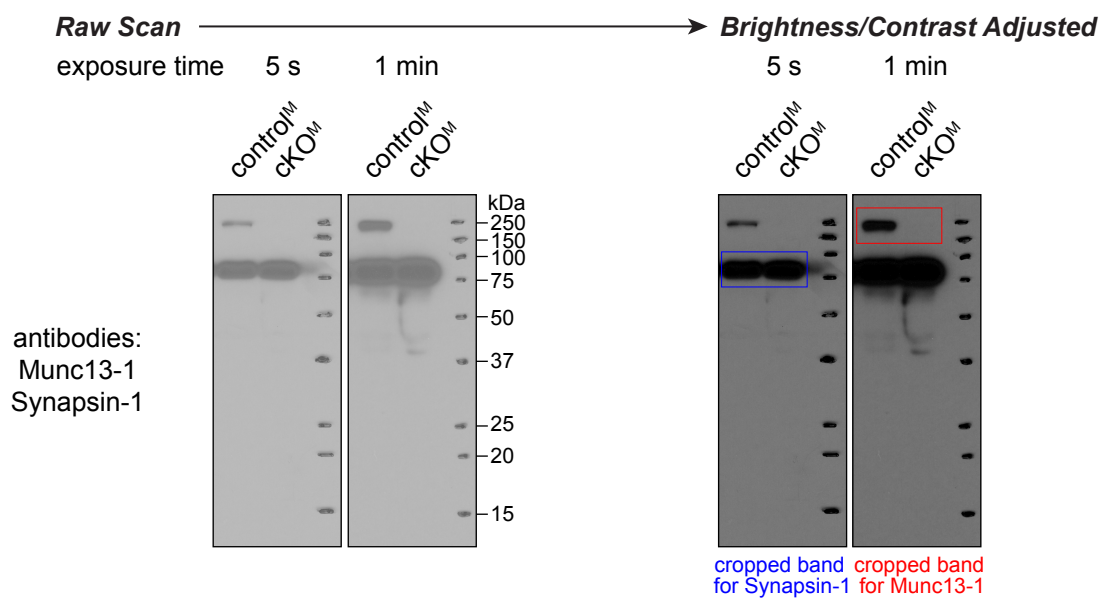

Tan et al., Figure 1 - figure supplement 3 - source data 1D

Supplement: Figure 1—figure supplement 3—source data 1. — (A) Original scans (1 s and 30 s) of Western blots shown in Figure 1—figure supplement 3C. (B) Raw grayscale scans (left) and brightness- and contrast-adjusted scans (right) of Western blots shown in Figure 1—figure supplement 3C. (C) Original scans (5 s and 1 min) of Western blots shown in Figure 1—figure supplement 3F. (D) Raw grayscale scans (left) and brightness- and contrast-adjusted scans (right) of Western blots shown in Figure 1—figure supplement 3F. [file elife-79077-fig1-figsupp3-data1.zip › Figure 1-figure supplement 3-source data 1/Figure 1-figure supplement 3-source data 1D-v1.pdf]

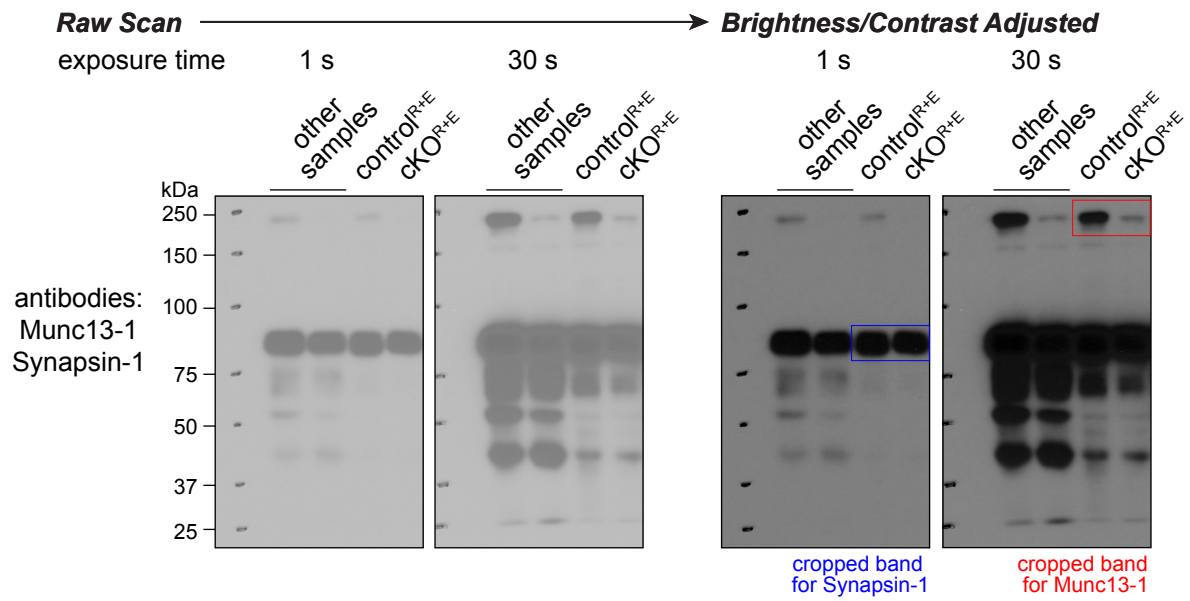

Tan et al., Figure 1 - figure supplement 3 - source data 1B

Supplement: Figure 1—figure supplement 3—source data 1. — (A) Original scans (1 s and 30 s) of Western blots shown in Figure 1—figure supplement 3C. (B) Raw grayscale scans (left) and brightness- and contrast-adjusted scans (right) of Western blots shown in Figure 1—figure supplement 3C. (C) Original scans (5 s and 1 min) of Western blots shown in Figure 1—figure supplement 3F. (D) Raw grayscale scans (left) and brightness- and contrast-adjusted scans (right) of Western blots shown in Figure 1—figure supplement 3F. [file elife-79077-fig1-figsupp3-data1.zip › Figure 1-figure supplement 3-source data 1/Figure 1-figure supplement 3-source data 1B-v1.pdf]

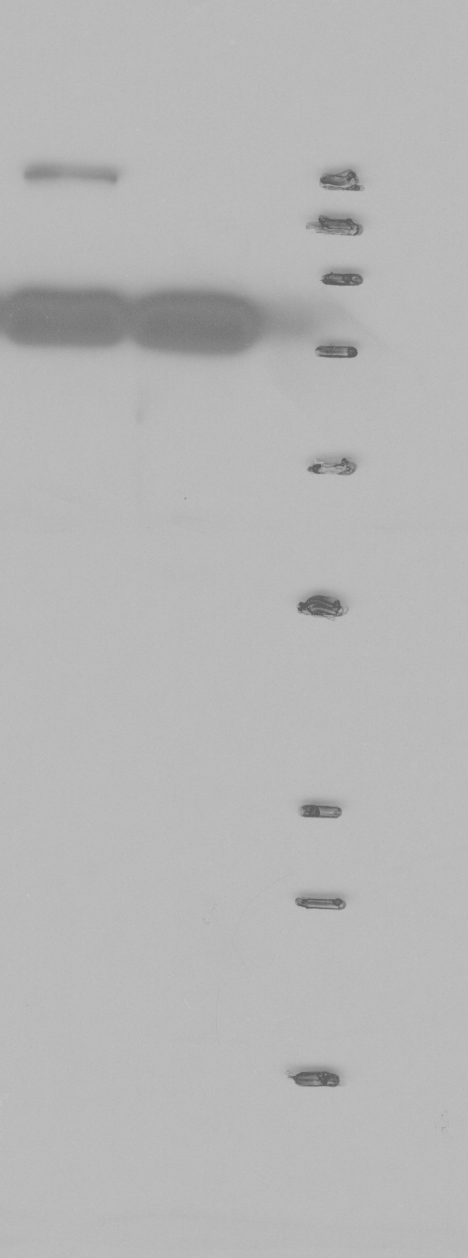

Supplement: Figure 1—figure supplement 3—source data 1. — (A) Original scans (1 s and 30 s) of Western blots shown in Figure 1—figure supplement 3C. (B) Raw grayscale scans (left) and brightness- and contrast-adjusted scans (right) of Western blots shown in Figure 1—figure supplement 3C. (C) Original scans (5 s and 1 min) of Western blots shown in Figure 1—figure supplement 3F. (D) Raw grayscale scans (left) and brightness- and contrast-adjusted scans (right) of Western blots shown in Figure 1—figure supplement 3F. [file elife-79077-fig1-figsupp3-data1.zip › Figure 1-figure supplement 3-source data 1/Figure 1-figure supplement 3-source data 1C-5s.tif]

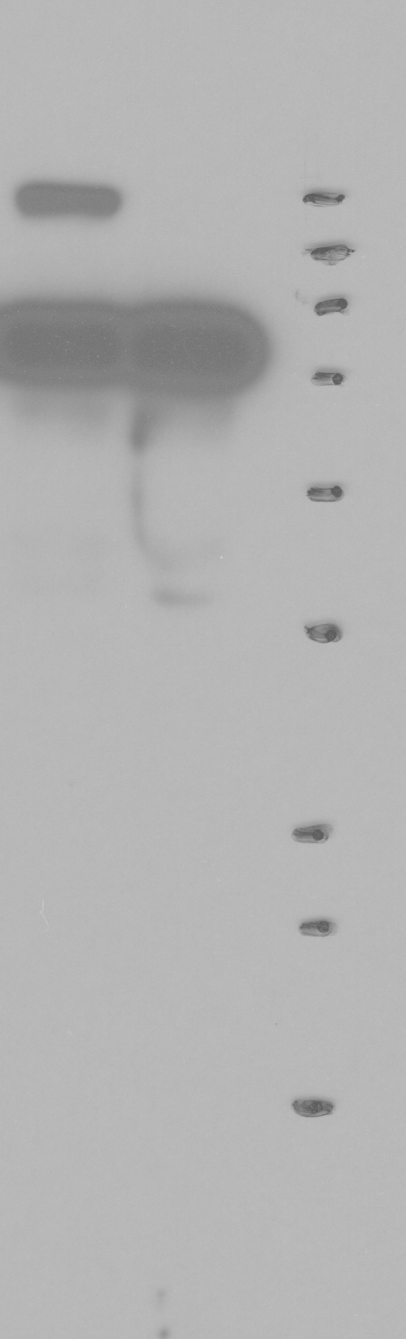

Supplement: Figure 1—figure supplement 3—source data 1. — (A) Original scans (1 s and 30 s) of Western blots shown in Figure 1—figure supplement 3C. (B) Raw grayscale scans (left) and brightness- and contrast-adjusted scans (right) of Western blots shown in Figure 1—figure supplement 3C. (C) Original scans (5 s and 1 min) of Western blots shown in Figure 1—figure supplement 3F. (D) Raw grayscale scans (left) and brightness- and contrast-adjusted scans (right) of Western blots shown in Figure 1—figure supplement 3F. [file elife-79077-fig1-figsupp3-data1.zip › Figure 1-figure supplement 3-source data 1/Figure 1-figure supplement 3-source data 1C-1min.tif]

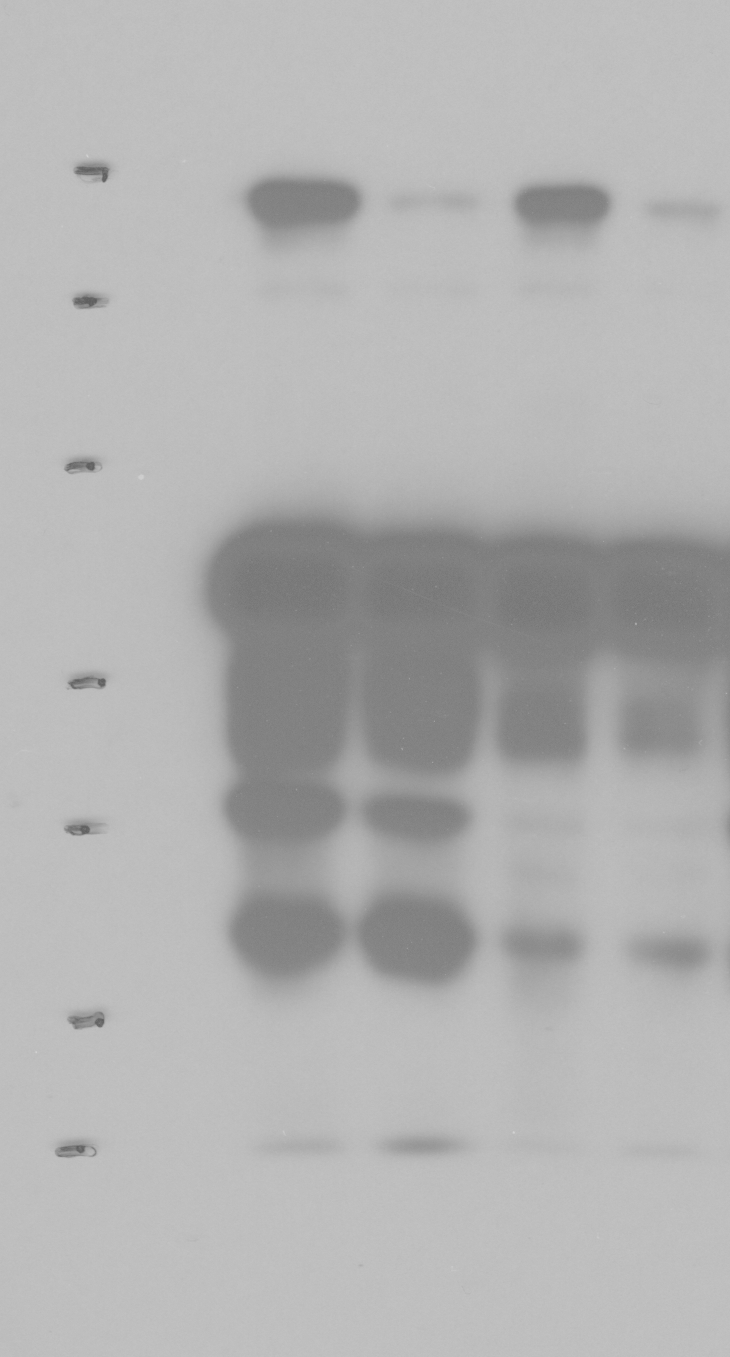

Supplement: Figure 1—figure supplement 3—source data 1. — (A) Original scans (1 s and 30 s) of Western blots shown in Figure 1—figure supplement 3C. (B) Raw grayscale scans (left) and brightness- and contrast-adjusted scans (right) of Western blots shown in Figure 1—figure supplement 3C. (C) Original scans (5 s and 1 min) of Western blots shown in Figure 1—figure supplement 3F. (D) Raw grayscale scans (left) and brightness- and contrast-adjusted scans (right) of Western blots shown in Figure 1—figure supplement 3F. [file elife-79077-fig1-figsupp3-data1.zip › Figure 1-figure supplement 3-source data 1/Figure 1-figure supplement 3-source data 1A-30s.tif]

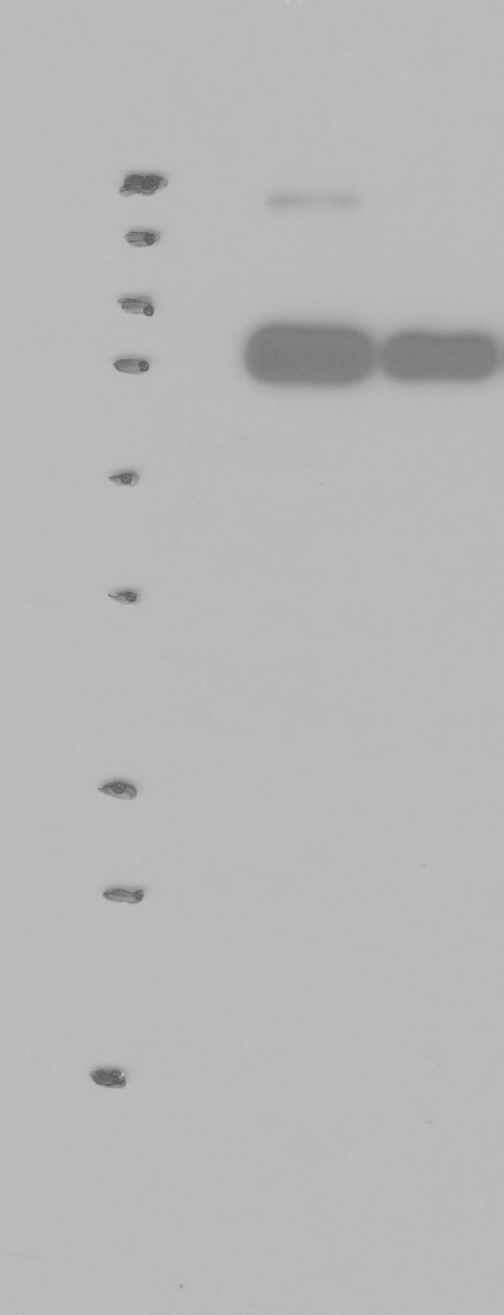

Supplement: Figure 2—figure supplement 1—source data 1. — (A) Original scans (5 s and 1 min) of Western blots shown in Figure 2—figure supplement 1F. (B) Raw grayscale scans (left) and brightness- and contrast-adjusted scans (right) of Western blots shown in Figure 2—figure supplement 1F. [file elife-79077-fig2-figsupp1-data1.zip › Figure 2-figure supplement 1-source data 1/Figure 2-figure supplement 1-source data 1A-5s.tif]

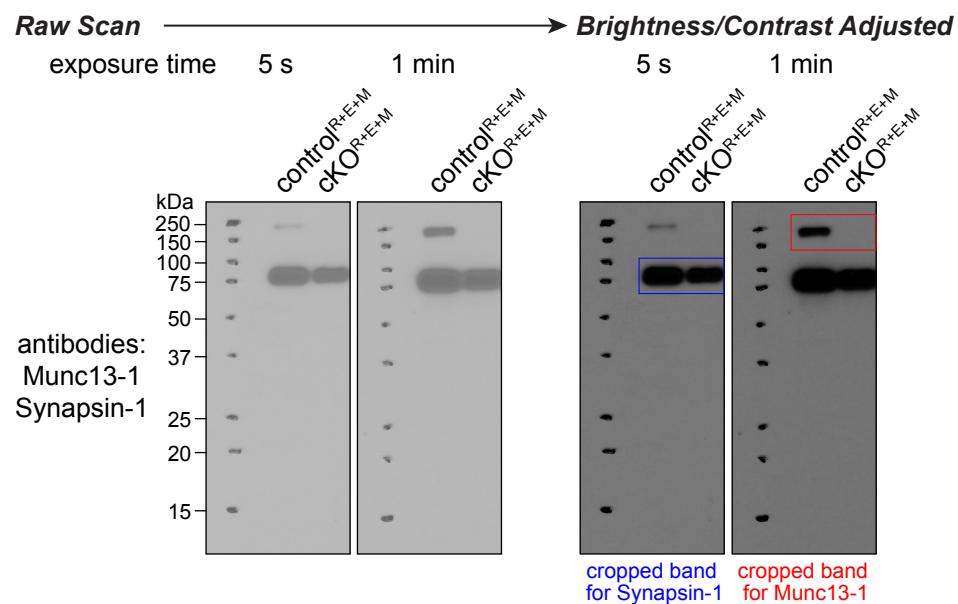

Tan et al., Figure 2 - figure supplement 1 - source data 1

Supplement: Figure 2—figure supplement 1—source data 1. — (A) Original scans (5 s and 1 min) of Western blots shown in Figure 2—figure supplement 1F. (B) Raw grayscale scans (left) and brightness- and contrast-adjusted scans (right) of Western blots shown in Figure 2—figure supplement 1F. [file elife-79077-fig2-figsupp1-data1.zip › Figure 2-figure supplement 1-source data 1/Figure 2-figure supplement 1-source data 1B-v1.pdf]

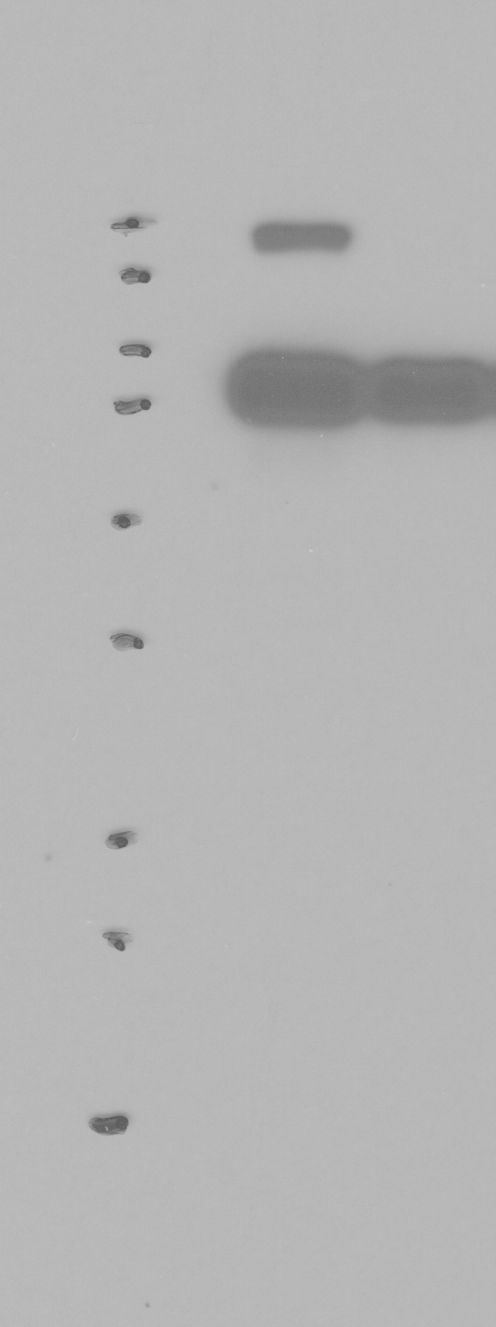

Supplement: Figure 2—figure supplement 1—source data 1. — (A) Original scans (5 s and 1 min) of Western blots shown in Figure 2—figure supplement 1F. (B) Raw grayscale scans (left) and brightness- and contrast-adjusted scans (right) of Western blots shown in Figure 2—figure supplement 1F. [file elife-79077-fig2-figsupp1-data1.zip › Figure 2-figure supplement 1-source data 1/Figure 2-figure supplement 1-source data 1A-1min.tif]

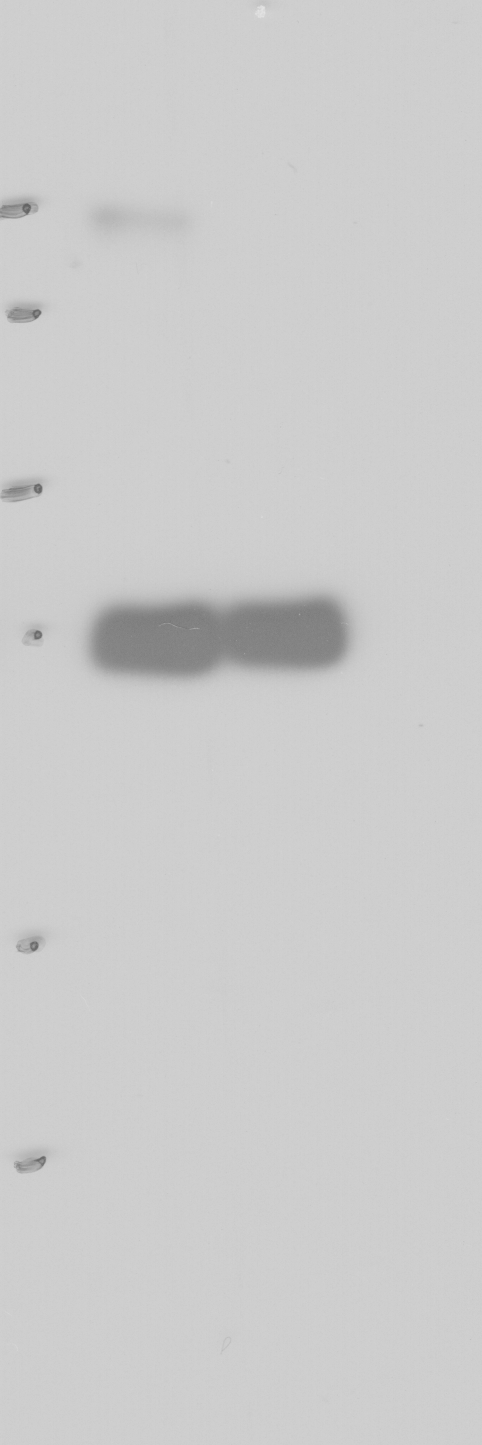

Supplement: Figure 2—figure supplement 2—source data 1. — (A) Original scans (1 s and 30 s) of Western blots shown in Figure 2—figure supplement 2F. (B) Raw grayscale scans (left) and brightness- and contrast-adjusted scans (right) of Western blots shown in Figure 2—figure supplement 2F. [file elife-79077-fig2-figsupp2-data1.zip › Figure 2-figure supplement 2-source data 1/Figure 2-figure supplement 2-source data 1A-1s.tif]

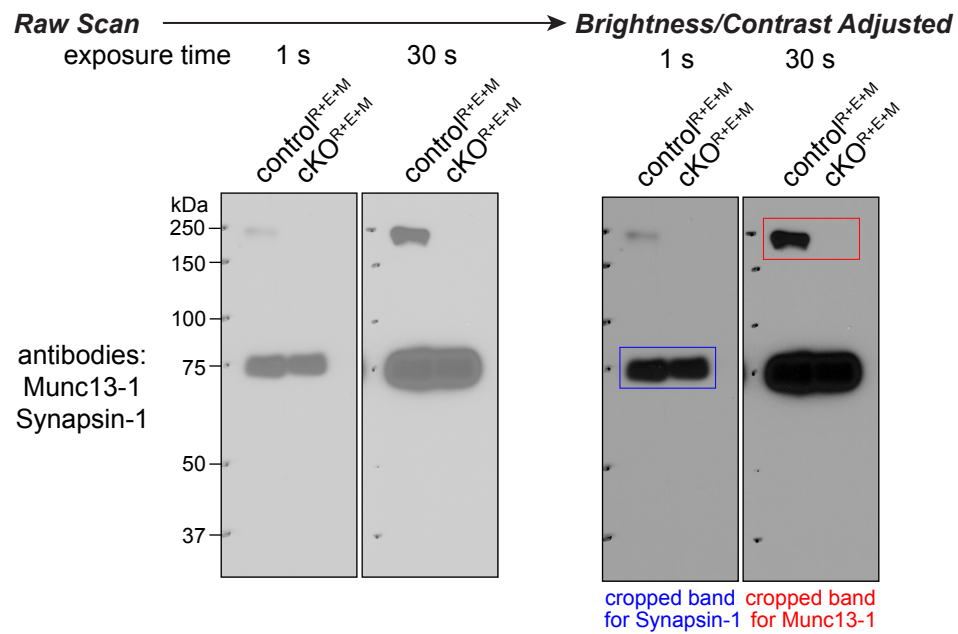

Tan et al., Figure 2 - figure supplement 2 - source data 1

Supplement: Figure 2—figure supplement 2—source data 1. — (A) Original scans (1 s and 30 s) of Western blots shown in Figure 2—figure supplement 2F. (B) Raw grayscale scans (left) and brightness- and contrast-adjusted scans (right) of Western blots shown in Figure 2—figure supplement 2F. [file elife-79077-fig2-figsupp2-data1.zip › Figure 2-figure supplement 2-source data 1/Figure 2-figure supplement 2-source data 1B-v1.pdf]

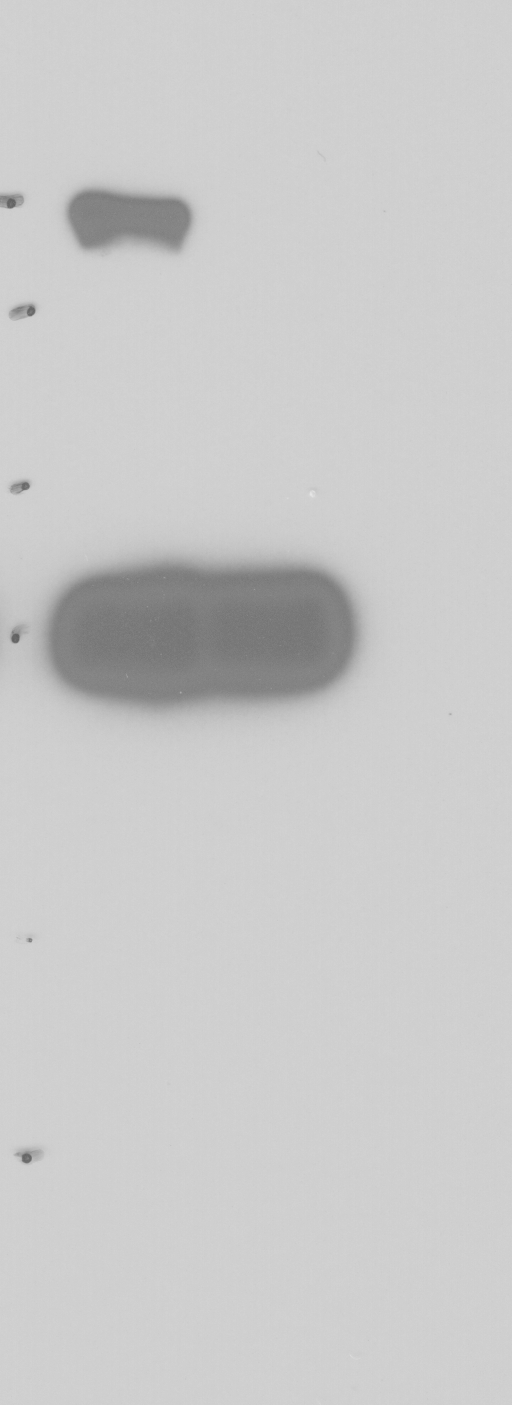

Supplement: Figure 2—figure supplement 2—source data 1. — (A) Original scans (1 s and 30 s) of Western blots shown in Figure 2—figure supplement 2F. (B) Raw grayscale scans (left) and brightness- and contrast-adjusted scans (right) of Western blots shown in Figure 2—figure supplement 2F. [file elife-79077-fig2-figsupp2-data1.zip › Figure 2-figure supplement 2-source data 1/Figure 2-figure supplement 2-source data 1A-30s.tif]
